# Human cytomegalovirus hijacks the autophagic machinery and LC3 homologs in order to optimize cytoplasmic envelopment of mature infectious particles

**DOI:** 10.1038/s41598-019-41029-z

**Published:** 2019-03-14

**Authors:** Clémence Taisne, Marion Lussignol, Eva Hernandez, Arnaud Moris, Lina Mouna, Audrey Esclatine

**Affiliations:** 1 Institute for Integrative Biology of the Cell (I2BC), CEA, CNRS, Univ. Paris-Sud, Université Paris-Saclay, 91198 Gif-sur-Yvette cedex, France; 2 Sorbonne Université, Centre d’Immunologie et des Maladies Infectieuses (CIMI-Paris), INSERM U1135, CNRS ERL 8255 Paris, France; 3 Virologie, APHP, Hôpital Paul Brousse, 94800 Villejuif, France

## Abstract

During its life cycle, Human cytomegalovirus (HCMV) tightly modulates autophagy, a vesicular pathway allowing degradation and recycling of cellular components. To study the interplay between autophagy and the viral life cycle, we established various autophagy-deficient human fibroblastic cell lines. By knocking down the expression or activity of five autophagy-related proteins, we confirmed the proviral function that the autophagic machinery exerts on HCMV production. Using 3D reconstruction from confocal microscopy and electron microscopy, we demonstrated that lipidated LC3-positive vesicles accumulated at the viral assembly compartment (vAC). The vAC is a juxtanuclear ring-shaped structure containing several organelles and membranes, where assembly and final envelopment of HCMV particles occur. Two LC3 homologs, GABARAPL1 and GATE16, also accumulated during HCMV infection and were associated with the vAC, in proximity with fragmented Golgi stacks. Additionally, we observed the formation of a pre-assembly compartment (PrAC) in infected cells, which consists of a juxtanuclear structure containing both fragmented Golgi and LC3-positive vesicles. Finally, we showed that highly purified extracellular viral particles were associated with various autophagy proteins. Our results thus suggest that autophagy machinery participates to the final cytoplasmic envelopment of HCMV viral particles into the vAC and that autophagy-related proteins can be spotted in the virions.

## Introduction

Human cytomegalovirus (HCMV) is one of the 8 Herpesviruses that can specifically infect humans, along with Herpes Simplex virus type 1 (HSV-1), Epstein-Barr virus (EBV) or Varicella Zoster virus (VZV). Its genome consists of a large double-stranded DNA, protected by an icosahedral capsid, surrounded by a tegument formed of viral phosphoproteins and an envelope derived from cell membranes decorated with viral glycoproteins^1^. HCMV infects many cell types, such as endothelial cells, macrophages or epithelial cells but its replication cycle is primarily studied in human fibroblasts. In these cells, HCMV enters the cytoplasm by fusion with the plasma membrane and its nucleocapsid (NC) is targeted to the nucleus using the microtubule network. The viral genome is introduced into the nucleus through nuclear pores, transcribed in a temporal pattern and immediate-early, early and late viral proteins are sequentially expressed in the cytoplasm. Capsid and tegument proteins are transported within the nucleus where NC assembly occurs. NCs surrounded by tegument proteins then translocate to the cytoplasm by transient wrapping with the inner nuclear membrane and fusion with the outer one. Simultaneously, the nucleus enlarges and adopts a kidney shape characteristic of HCMV-infected cells^2,3^. The viral assembly compartment (vAC), a structure specific to HCMV is housed in the area of the cytoplasm delimited by the nucleus indentation^4,5^. The vAC is composed of a set of vesicles organized concentrically around a microtubule organizing center (MTOC) and is a consequence of a drastic rearrangement of the secretory and endocytic organelles within the cytoplasm^6^. Early endosomes, surrounded by Trans Golgi Network (TGN) are found in the inner part of the vAC, while Golgi stacks form a ring at the outer part of the structure^7^. Markers of late endosomes are also found in the vAC while endoplasmic reticulum (ER) and mitochondria are excluded. Viral tegument and envelope proteins accumulate in the vAC allowing final tegumentation and envelopment of NCs released from the nucleus. The exact composition of HCMV envelopes is still discussed: although it is accepted that these are TGN membranes, studies show that other vesicular membranes can be used^5,8^. Both the mechanism of vAC formation and the acquisition of viral final envelope are still not clearly elucidated. Finally, enveloped viruses surrounded by vesicles travel to the plasma membrane, where they exit the cell by exocytosis. In fibroblasts, the entire process of the viral cycle is long and lasts for 4 to 5 days.

We previously studied the relationships between HCMV and a vesicular process that degrades and recycles many cellular components and organelles, named autophagy^9–11^. The autophagic vesicles, or autophagosomes, are double-membrane structures in charge of capturing cytoplasmic cargos^12^. The autophagosome arises from a phagophore, a transient cup-shaped double-membrane structure, which gradually elongates and seals to constitute the autophagosome. From the formation of the phagophore to the fusion of the autophagosome with the lysosome, autophagy requires dozens of AuTophaGy-related (ATG) proteins which were initially identified by genetic analysis in yeast. LC3, a mammalian homolog of yeast ATG8, can be conjugated with a lipid, phosphatidylethanolamine (PE) thanks to several ATG proteins to form LC3-PE, also called LC3-II. The ubiquitin-like conjugation system of LC3 requires an E3-like ATG5-ATG12/ATG16L1 complex. A second conjugation system comprising the E1-like ATG7 and E2-like ATG10 allows the conjugation of ATG5 with ATG12^13^. LC3-II mediates several functions, elongation and/or sealing of phagophores but also recognition of selective cargoes through autophagic receptors such as p62/SQSTM1. Human cells encode several ATG8 homologs, divided into two subfamilies: LC3, which includes LC3A B and C, and GABARAP, which includes GABARAPL1 and GATE16 (GABARAPL2), and all of them can be lipidated^14^. To be conjugated with PE, ATG8 homologs need to be first processed by ATG4B, the sole protease among ATG proteins, exposing a glycine residue at their C-terminus^15^. The second role of ATG4B is to hydrolyze lipids from LC3 (and its homologs) to recycle it into LC3-I. Autophagy is highly regulated by several key complexes. The complex containing ULK1, ATG13, FIP200 and ATG101 is essential for membrane nucleation and, in response to stress, it activates the complex containing ATG14, BECN1 and VPS34, which promotes autophagosome formation^16,17^. A second BECN1 complex is involved in autophagosome maturation. It is also important to mention that lipidated LC3 can be inserted into single membrane compartments of the endolysosomal system, including LC3-associated phagocytosis, macropinocytosis or entosis^18,19^. These processes are referred to as non-canonical autophagy and in that case, LC3 lipidation is independent from the upstream autophagic regulators, such as ULK1.

We and others have observed that HCMV is able to regulate autophagy throughout the viral cycle^11,20,21^. It initially activates the formation of autophagosomes to secondly block the fusion of autophagosomes with lysosomes and thus blocks the degradation step^10^. HCMV thus inhibits the so-called autophagic flux, leading to an accumulation of non-degraded LC3 during viral infection^11^. At least two viral proteins, IRS1 and TRS1, are involved in inhibiting autophagy, whereas early autophagy activation could be related to the presence of the viral genome in infected cells^11,20^. We previously reported that pharmacological induction of autophagy leads to an increased viral production while its inhibition decreases HCMV production^11^. Autophagy thus exerts a proviral activity. However, the specific part(s) of the viral cycle favored by autophagy is currently unknown as well as the step of autophagy (i.e. initiation, maturation, and degradation) required to mediate this enhancement of HCMV production.

In this work, we aimed at studying the impact of autophagy on the different stages of HCMV multiplication using different cell lines deficient in autophagy. Invalidation of several autophagy proteins makes it possible to differentiate the specific role of a particular ATG protein or of the whole autophagic process.

## Results

### Knockdown of autophagy proteins decreases production of infectious viral particles

**Figure 1:**
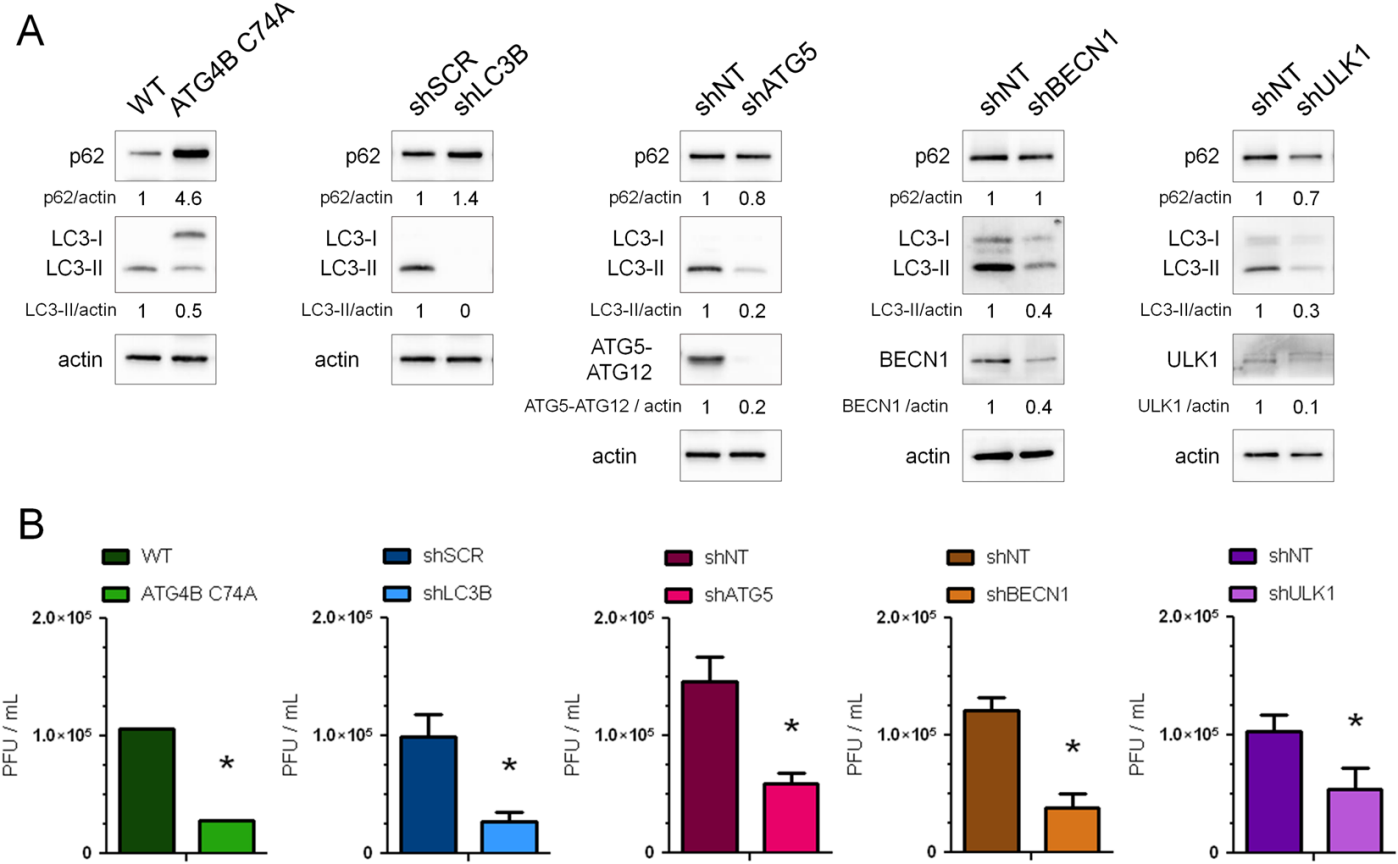
Several autophagic proteins are beneficial for HCMV production. Stable HFF cell lines either expressing a dominant-negative mutant of ATG4B or knockdown for the indicated autophagic proteins (LC3B, ATG5, BECN1 or ULK1) and their corresponding control cell lines (WT: wild type HFF, shSCR: scrambled shRNA, shNT: non-target shRNA) have been used. (**A**) Immunoblot analysis of p62, LC3B, ATG5, BECN1 and ULK1 expression confirms the inhibition of autophagy in the indicated cell lines. Actin was used as a loading control. Full-length blots are presented in Fig. S7 (**B**) Viral titers were determined 10 days post-infection with HCMV AD169 strain at MOI 0.01 in the different cell lines. Error bars indicate SEM from at least three independent experiments. *p < 0.05 (Student’s t test).

We have previously shown that pharmacological inhibition of autophagy reduced HCMV production^11^. In order to define the effector(s) involved in this process, we studied HCMV multiplication in human foreskin fibroblasts (HFF) invalidated for different autophagy proteins. To this purpose, we first established cell lines stably expressing a *trans-*dominant negative form of ATG4B (ATG4B C74A) or shRNA targeting the expression of LC3B (shLC3B), ATG5 (shATG5), BECN1 (shBECN1) and ULK1 (shULK1), using transduction with different lentiviral vectors as described in the Methods section^22^. Inhibition of autophagy and protein silencing were confirmed in the different cell lines (Fig. 1A). The inhibitory activity of ATG4B C74A was demonstrated by accumulation of the non lipidated form of LC3 (LC3-I) because ATG4B C74A both impairs LC3 lipidation (LC3-II formation) and sequesters LC3 from cytosol^23^. Moreover, cell proliferation was not significantly impacted by the knockdown of autophagy proteins (Supplementary Fig. S1). In order to evaluate viral production in these different cell lines, cells were infected with HCMV at a multiplicity of infection (MOI) of 0.01 during 10 days and viral titers were evaluated in the supernatant (Fig. 1B). We observed a decreased production of HCMV in all the autophagy-deficient cell lines tested. Viability of cells infected in the same conditions at the same MOI was not significantly affected by the knockdown of autophagy proteins (Supplementary Fig. S1).

In order to test whether the effect of autophagy was not restricted to the HCMV strain we used (AD169), we also infected cells with TB40/E HCMV strain, which has a broad cell tropism^24^. In ATG4B C74A HFF, we observed a similar decrease in TB40/E viral production (Supplementary Fig. S1), showing a beneficial effect of autophagy independently of the viral strain used. These results demonstrate that several components of the autophagic process are required to improve HCMV production.

### LC3-positive vesicles accumulate in the viral assembly compartment

**Figure 2:**
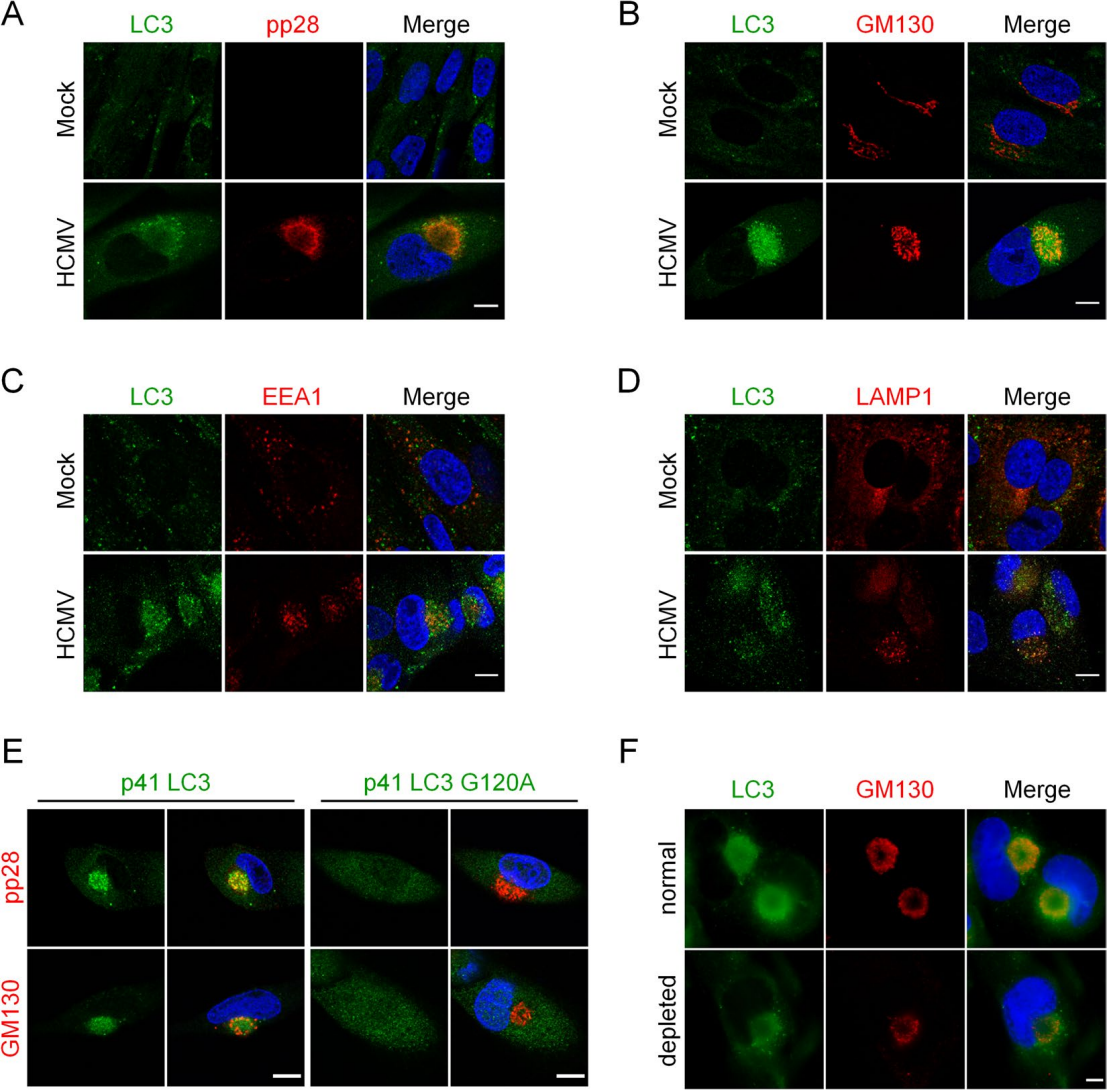
Vesicles containing lipidated form of LC3 accumulate in the vAC. Confocal images of HFF mock-infected (Mock) or infected with HCMV AD169 strain at MOI 0.5 for 4 days and immunostained for LC3 and (**A**) pp28 (viral tegument protein), (**B**) GM130 (Golgi marker), (**C**) EEA1 (early endosome marker), or (**D**) LAMP1 (late endosome/lysosome marker). Representative images of 3 independent experiments. See also supplementary videos S2-4. (**E**) Confocal images of cells stably expressing p41-LC3 or p41-LC3 G120A infected with HCMV at MOI 0.5 for 4 days and immunostained for exogenous LC3 with pp28 or GM130. (**F**) Representative images of WT HFF infected with HCMV at MOI 0.5 for 4 days, and permeabilized either after fixation (“normal”) or before fixation (“depleted”) to eliminate soluble cytosolic proteins. Cells were immunostained for LC3 and GM130. Nuclei were subsequently stained with DAPI. Scale bar = 10 µm.

We had previously reported a progressive accumulation of LC3-II during HCMV infection^10^. In order to study the subcellular localization of LC3 during HCMV life cycle, we co-immunostained LC3 with pp28, a tegument viral protein or with GM130, a Golgi marker, (Fig. 2A,B respectively), which both localize within the viral assembly compartment (vAC) during infection^3,4,6^. We clearly observed an accumulation of LC3 puncta within this juxtanuclear compartment four days post-infection (pi). In contrast, LC3 puncta were homogenously distributed in the cytoplasm of mock-infected cells (Fig. 2). Three-dimensional reconstructions from z-series confocal microscopy images confirmed the accumulation of LC3 puncta mainly in a ring-shaped structure centered on the vAC with partial colocalization with pp28 and minimal colocalization with Golgi fragments (Supplementary Information S2,S3). We noticed that the vAC was located in the hollow of the characteristic kidney-shaped nucleus of infected cells (clearly visible in Fig. S3). Early endosomes, labeled with EEA1 (early endosome antigen 1), localized in the center of vAC in HCMV infected cells (Fig. 2C and Supplementary S4), as expected^7^, and were surrounded by LC3 puncta. Late endosomes/lysosomes (LAMP1-positive) were also observed within the vAC and partially colocalized with LC3 (Fig. 2D). Therefore, our studies suggest that LC3 puncta are present at the outer edge of the vAC in a ring-like pattern, interspersed with a Golgi marker. In order to explore whether LC3 puncta present inside the vAC were lipidated LC3 or not, we used two tagged versions of LC3, p41-LC3, which corresponds to the native form of LC3 and p41-LC3 G120A, which contains a well-characterized mutation of LC3 impeding its lipidation^22^. By immunostaining of either GM130 or pp28 to visualize the vAC and staining of exogenous LC3, we observed that LC3 G120A was homogenously distributed in the cytoplasm, whereas wild-type LC3 was present into the vAC (Fig. 2E). To confirm that LC3 was associated with membranes, HFF cells infected by HCMV were permeabilized before fixation to remove soluble proteins from the cytosol and immunostained for LC3 and vAC. LC3 staining was still visible in the vAC in these conditions (Fig. 2F), demonstrating that LC3 present in the vAC is associated with membranes. Taken together, our results clearly demonstrated that vAC contained LC3-II-positive vesicles.

**Figure 3:**
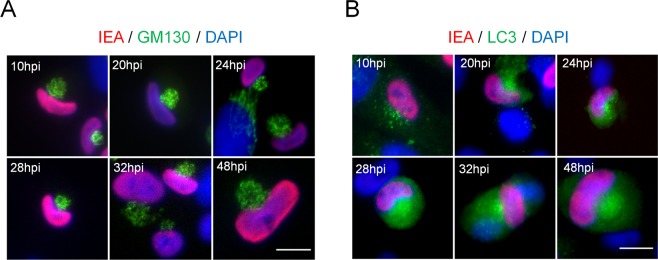
Formation of a pre-assembly compartment (PrAC) containing Golgi stacks and LC3 starts early in the viral life cycle. (**A**,**B**) Representative images of HFF infected with HCMV AD169 strain at MOI 1 during 10 to 48 hours and immunostained for IEA (immediate-early viral antigens) and for GM130 (**A**) or LC3 (**B**). Nuclei were subsequently stained with DAPI. Representative images of 2 independent experiments. Scale bars = 10 µm.

We then performed time-course experiments to determine when LC3-positive vesicles start to accumulate in the area close to the nucleus after HCMV infection (Fig. 3). To identify infected cells, we used an antibody directed against immediate-early antigens IE1 and IE2 (nuclear localization), since pp28 is expressed later and therefore would not be detected at early times of infection. In HCMV-infected cells, we observed that modifications of the nucleus shape occurred as early as 10 hours pi. At this time point, LC3 also started to accumulate in the hollow of the nucleus in proximity with the Golgi marker (Fig. 3A). Between 24 and 48 hours pi, LC3 began to localize in a pre-assembly compartment (PrAC) together with Golgi fragments.

### Autophagy does not play a role in HCMV infection before assembly of viral particles

**Figure 4:**
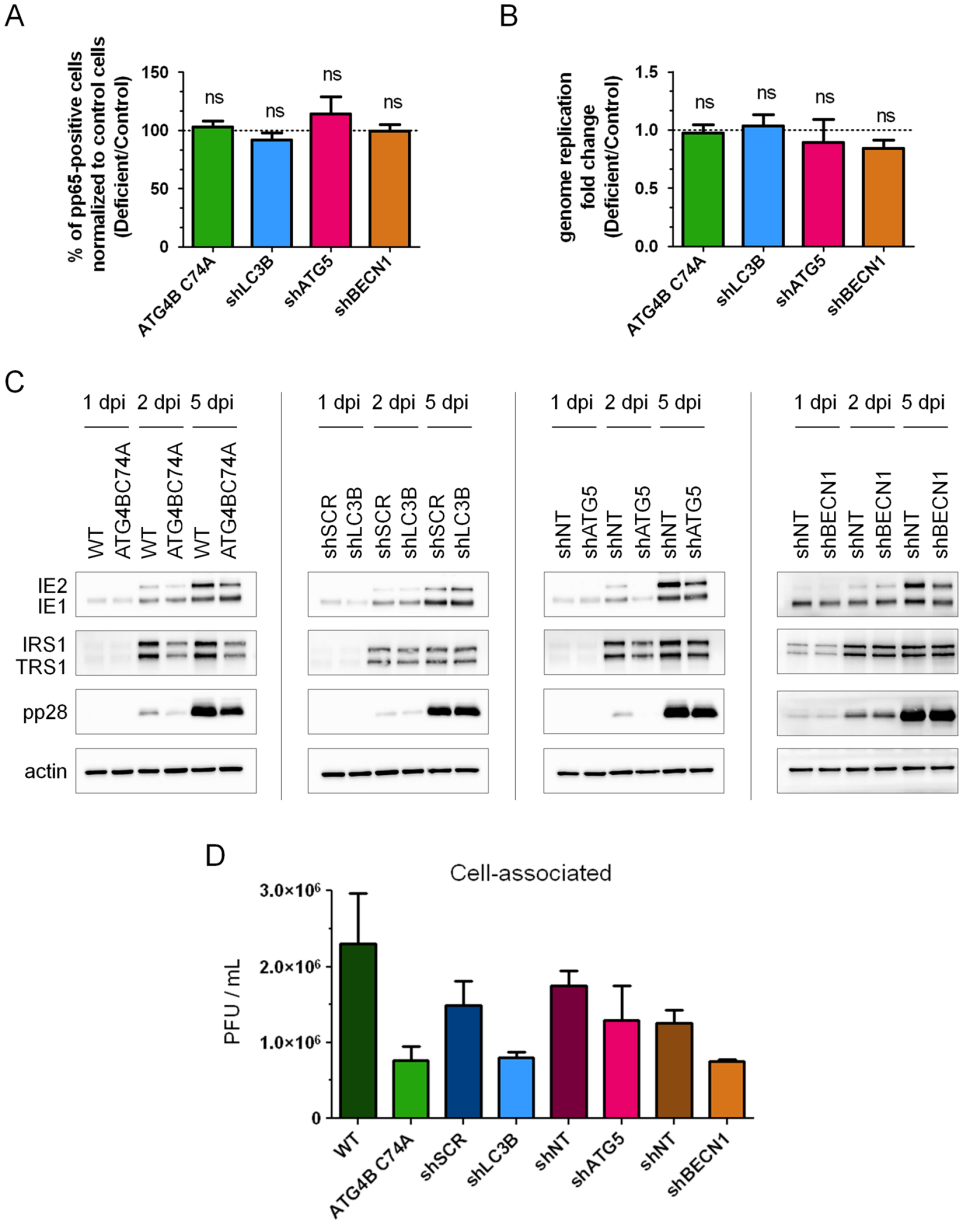
Roles of the autophagy machinery in different steps of the viral cycle. (**A**) Quantification of HCMV entry by immunofluorescence detection of the viral tegument protein pp65 2 h after contact with the virus (MOI 1). DAPI was used to stain nuclei. Percentages of pp65-positive cells correspond to viral entry into the indicated cell lines compared to control cells (deficient: Autophagy-deficient). (**B**) Quantification of HCMV genome replication 4 dpi at MOI 0.5 by real time PCR in the indicated cell lines, compared to control cells. (**C**) Expression of several viral proteins after HCMV infection of control and autophagy-deficient cell lines at MOI 0.5 for 1, 2 or 5 days post infection (dpi). Actin was used as a loading control. Full-length blots are presented in Fig. S7. (**D**) Indicated control and deficient cells were infected at MOI 0.5 with HCMV AD169 strain. Cell-associated viruses were collected 4 dpi and the amount of infectious virus was measured. Results represent the mean values of 3 independent experiments. ns: non-significant (Student’s t test).

Impact of autophagy deficiency in different steps of HCMV life cycle was studied using cells stably expressing ATG4B C74A or cells expressing shLC3B, shATG5 or shBECN1 (Fig. 4). The appearance in the nucleus of the tegument protein pp65, which is contained in the incoming viral particles, can be used to monitor viral entry^25^. We infected cells with HCMV for 2 hours and labelled for pp65 and DAPI to determine the percentage of pp65-positive cells (Fig. 4A). No significant difference in viral entry was noted between the different cell lines. Using qPCR, we also quantified viral genome accumulation four days pi, and observed that knocking down the expression of autophagy proteins such as LC3B, ATG5, BECN1 or ATG4B did not modulate viral genome replication (Fig. 4B). We also monitored the expression of viral proteins IE1/IE2, TRS1/IRS1 and pp28 in different deficient cell lines (Fig. 4C). Globally, we did not observe a clear impact on viral protein expression, although we noticed a slight decrease of pp28 expression in the different cell lines and a decreased expression of TRS1 and IRS1 in ATG4B C74A cells. Extension of our analysis to 7 days pi (Supplementary Fig. S5) suggested that expression of IRS1/TRS1 was only delayed in ATG4B C74A cells. Finally, we evaluated the impact of autophagy on viral release from fibroblasts by titration of cell-associated viral particles in single step growth experiments (Fig. 4D). We observed that viral titers are lower in deficient cells, demonstrating that the role of autophagy precedes the exit of HCMV by exocytosis. Taken together, our results suggested that the autophagic machinery did not significantly participate in viral entry, genome replication, viral protein expression and viral release.

### ATG8 homolog-positive vesicles accumulate in the vAC

**Figure 5:**
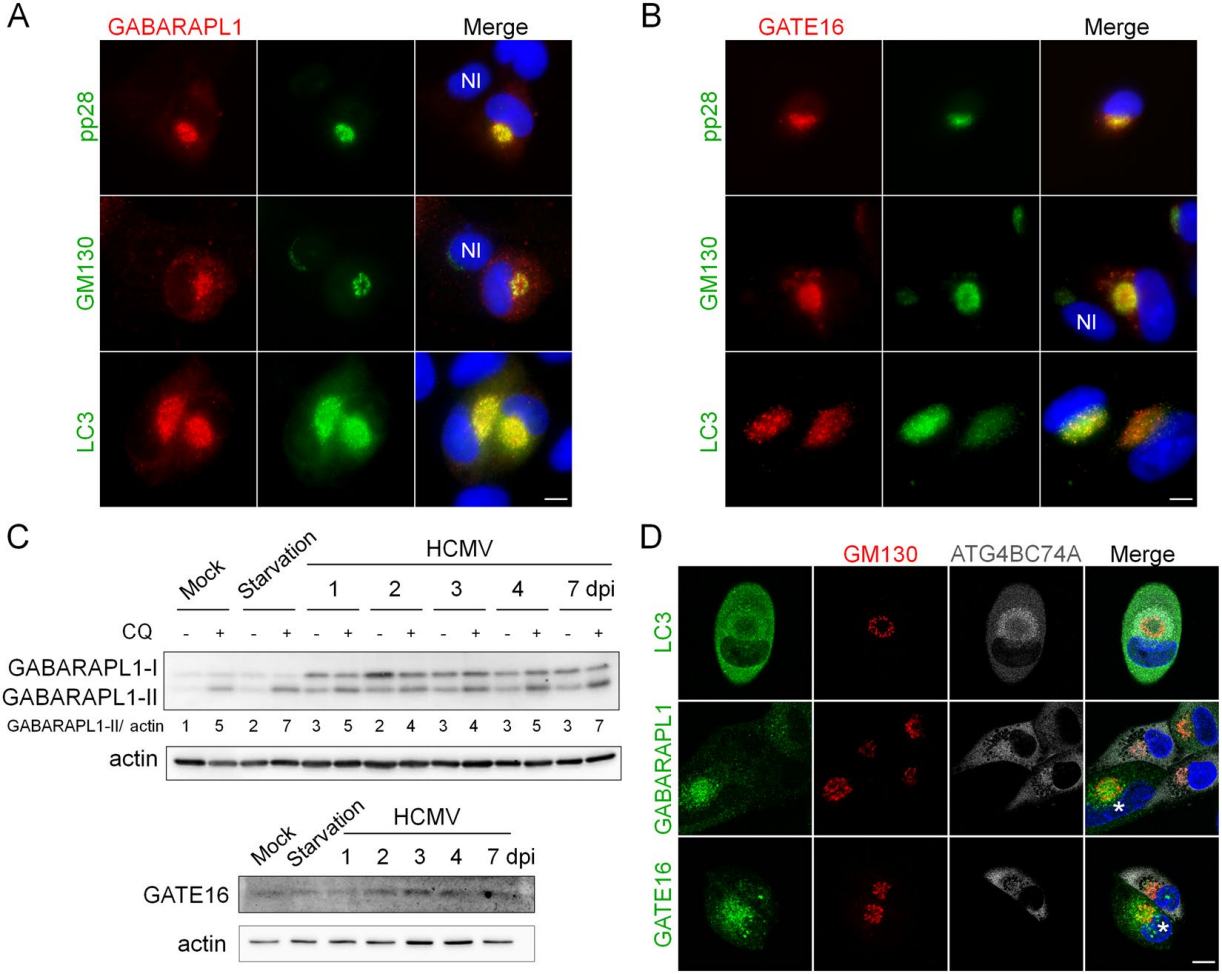
ATG8 homologs accumulate in the vAC in their lipidated form. (**A**,**B**) Representative images of HFF cells infected with HCMV AD169 strain at MOI 0.5 for 4 days and immunostained for (**A**) GABARAPL1, and pp28 (viral tegument protein), GM130 or LC3; (**B**) GATE16 and pp28, GM130 or LC3 (NI: non-infected cells) (**C**) Immunoblot analysis of GABARAPL1 and GATE16 expression in HCMV-infected cells at the indicated times after infection. Autophagic flux was measured by addition of chloroquine (CQ) 4 h before lysis. Actin was used as a loading control. Full-length blots are presented in Fig. S7 (**D**) Confocal images of autophagy-deficient cells stably expressing ATG4B C74A infected with HCMV at MOI 0.5 for 4 days and immunostained for GM130 (Golgi) with LC3 or GABARAPL1 or GATE16. (*) non-transduced cells. Nuclei were subsequently stained with DAPI. Scale bars = 10 µm.

Until now, we have focused our work on the subcellular localization of LC3 in infected cells. We then decided to investigate the fate of other mammalian ATG8 homologs, GABARAPL1 and GATE16 (GABARAPL2) during HCMV infection^14,26^. Co-immunostaining demonstrated that GABARAPL1 was found inside the vAC 4 days pi and colocalized with LC3 (Fig. 5A). Like previously, the vAC was labeled with GM130/pp28 staining. Similarly, GATE16 localized in the vAC after HCMV infection (Fig. 5B). Interestingly, in the same images, GATE16 and GABARAPL1 were almost undetectable in neighboring non-infected cells, suggesting an overexpression in HCMV-infected cells. Immunoblot analysis of GABARAPL1 and GATE16 expression confirmed that the proteins accumulated during HCMV infection, whereas their expression is less modified by starvation-induced autophagy (Fig. 5C). Treatment with chloroquine induced a limited increase of GABARAPL1-II in HCMV infected cells until 3 days post-infection, indicating a restricted autophagic flux.

Since ATG4B also participates in the processing of GABARAPs, we tried to determine whether GABARAPL1 and GATE16 present inside the vAC are lipidated or not, by using cells stably expressing ATG4B C74A, which are unable to lipidate any ATG8 homologs^27^. Cells were infected with HCMV for 4 days and labeled for GM130 and different ATG8 homologs (Fig. 5D). We observed an important accumulation of LC3 in infected cells but this pool of non-lipidated LC3 seems excluded from the vAC, confirming results obtained using LC3 G120A (Fig. 2E). A similar experiment showed that non-lipidated forms of GABARAPL1 and GATE16 were homogenously present in the cytoplasm of ATG4B C74A expressing cells, whereas they accumulated in the vAC in cells which did not express the dominant negative protein (Fig. 5D). Taken together, our results demonstrate that vesicles associated with different ATG8 homologs are part of the vAC during HCMV infection.

Interestingly, we still observed the formation of vAC in these deficient cells, labeled with GM130 (Fig. 5D), meaning that ATG4B is not necessary for the formation of vAC. Similarly, vAC was present in absence of LC3B or ATG5 (Supplementary Fig. S6). These results suggest that the autophagic process is not involved in the formation of vAC.

### Autophagy proteins are present in purified infectious extracellular HCMV

**Figure 6:**
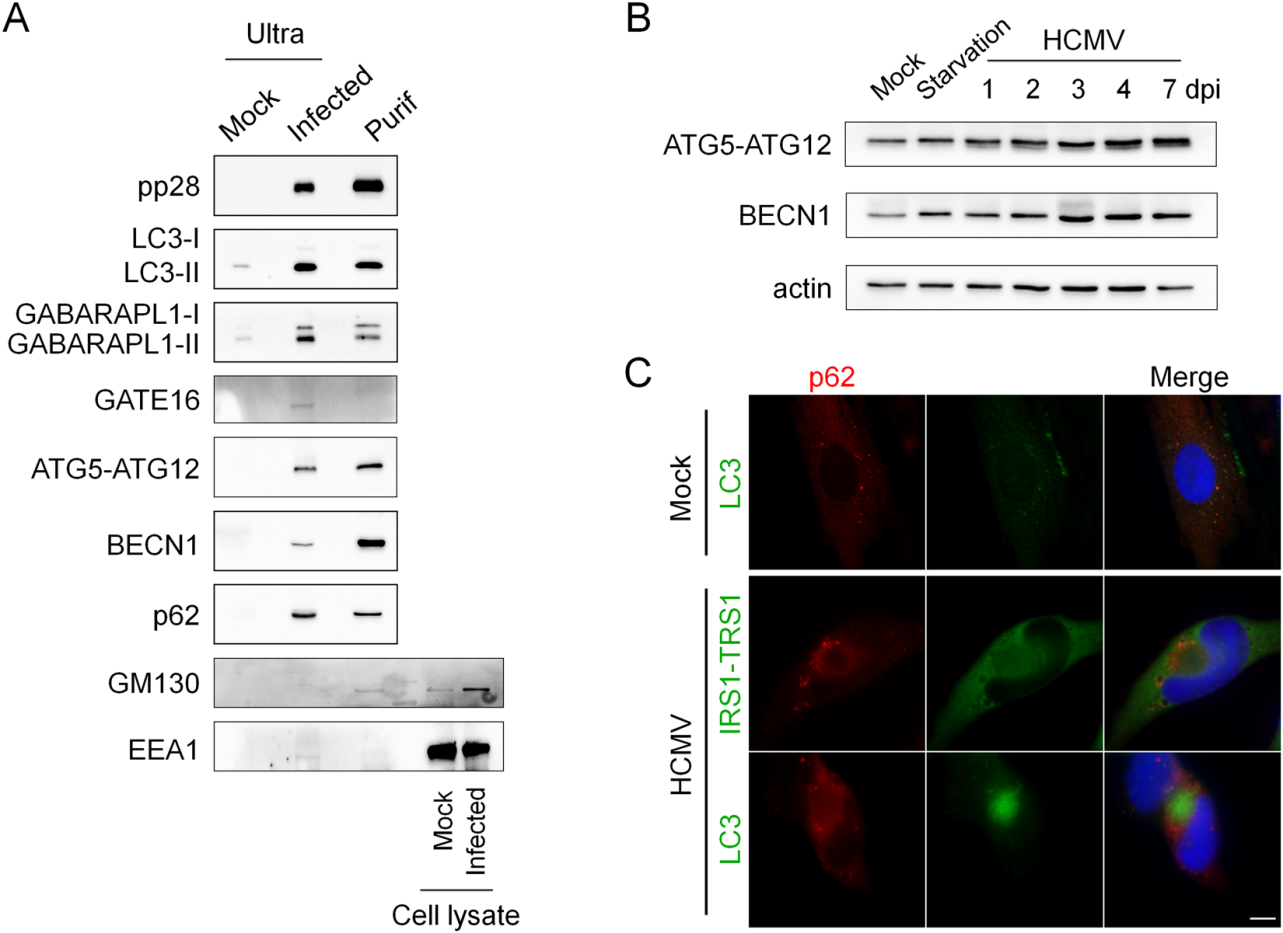
Autophagic proteins accumulate during infection and are present in extracellular viral particles. (**A**) Immunoblot analysis of cellular proteins contained in purified extracellular viral particles. Ultra (ultracentrifuged supernatant); Purif (purified virus by tartrate/glycerol gradient centrifugation). Supernatant of mock-infected cells was ultracentrifuged and used as a negative control and cell lysate are used as a positive control. (**B**) Immunoblot analysis of ATG5 and BECN1 expression in HCMV-infected cells at the indicated times after infection. Actin was used as a loading control. Full-length blots are presented in Fig. S7. (**C**) Representative images of HFF cells infected with HCMV AD169 strain at MOI 0.5 for 4 days and immunostained for p62 and IRS1/TRS1 (viral proteins) or LC3. Nuclei were subsequently stained with DAPI. Scale bars = 10 µm.

Given the localization of ATG8 homologs in the vAC, we hypothesized that HCMV uses autophagic membranes for the maturation of the viral particle and the acquisition of its final envelope in the vAC. If so, membrane-associated proteins such as ATG8 homologs should be incorporated into purified extracellular viral particles. To test this, we enriched viral material from cell-free supernatants of HCMV-infected human fibroblasts by ultracentrifugation and blotted virus pellets against the tegument protein pp28, autophagic proteins and membranous markers such as GM130 and EEA1. Supernatants from infected cells contain two classes of enveloped particles, HCMV virions and larger structures corresponding to non-infectious capsidless dense bodies^28^. As a negative control, we used supernatants from mock-infected cells enriched under the same conditions. We detected high levels of pp28 and LC3-II, and slightly detectable levels of LC3-I, associated with viral pellets (Fig. 6A). We also observed the presence of two forms of GABARAPL1 and one weak band of GATE 16 that we supposed to be the lipidated form. We looked for other proteins of the autophagic machinery and found that ATG5, BECN1 and the autophagy receptor p62 were pelleted with HCMV particles. All these different autophagy proteins were not detected (or at very low levels for LC3-II and GABARAPL1) in the supernatants of mock-infected cells. In order to exclude any association with cellular debris, exosomes and viral dense bodies, HCMV particles were further purified with a tartrate/glycerol gradient purification^29^ and we confirmed the presence of the same autophagy proteins associated with purified virions, except for GATE16 (Fig. 6A). To show the specificity of the association between autophagic proteins and HCMV particles, we also tested the presence of EEA1 and GM130. They are almost undetectable in both enriched supernatant of infected cells and purified viral fractions and only a small amount of Golgi marker was detected in the purified fraction, as previously described^8^. Since ATG5 and BECN1 are enriched with purified virions, we wanted to know whether they accumulated during HCMV infection. We found that their expression was modestly increased in HCMV infected cells (Fig. 6B). Since we had previously published that p62 accumulated during HCMV infection as a consequence of the autophagic flux inhibition^10^, we performed an immunostaining and observed a relocation of p62 during HCMV infection, mainly at the periphery of the vAC (Fig. 6C).

**Figure 7:**
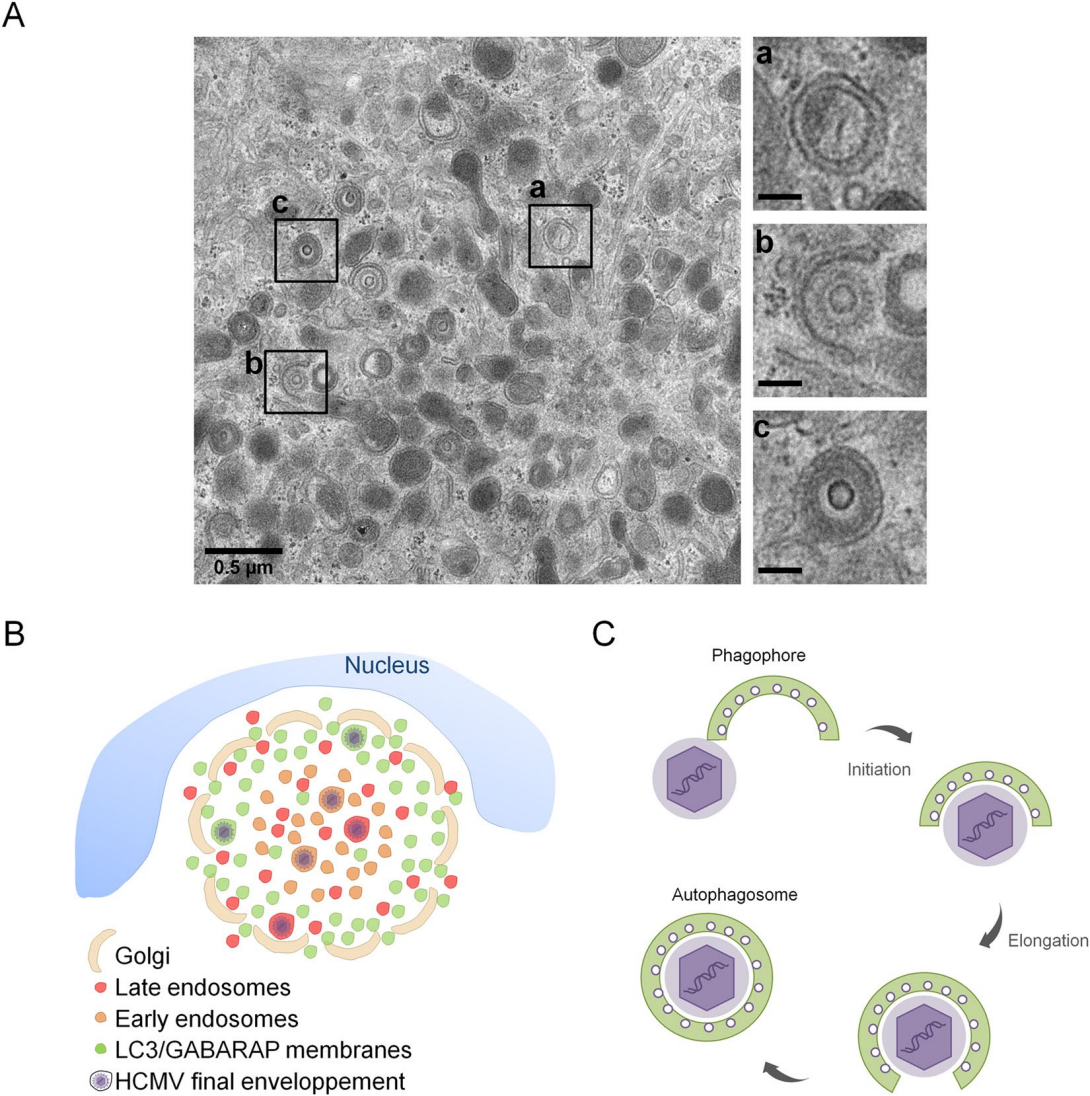
HCMV uses autophagic membranes for its assembly. (**A**) Transmission electron micrographs of HFF infected by HCMV at MOI 0.5 during four days. Inserts showed a) autophagosome, b) tegumented nucleocapsid envelopment c) enveloped virus in a vesicle. Insert scale bars = 0.1 µm. (**B**,**C**) Model based on our data and prior confocal and ultrastructural analyses. (**B**) Schematic representation of vAC structure and localization of LC3/GABARAP-positive vesicles in vAC with respect to cellular and viral components. HCMV could use membranes from different compartments and in particular autophagic vesicles for its final envelopment. (**C**) Schematic representation of HCMV envelopment. (1) The viral budding process involves a small double membrane crescent-shape structure which contains viral glycoproteins; (2) this double membrane elongates and fuses around the tegumented nucleocapsid to give rise to (3) an enveloped viral particle within a single-membrane vesicle. This process shares common points with phagophore and autophagosome formation.

Thus, our results suggest that ATG proteins are incorporated in the viral particle during its cytoplasmic envelopment inside the vAC. To go further, we used transmission electron microscopy (TEM) to visualize ultrastructure of the vAC and cytoplasmic HCMV envelopment (Fig. 7A). As expected from our immunofluorescence studies, we observed numerous double membrane vesicles that we considered as autophagosomes in the vAC. We also noted that these autophagosomes were smaller than expected (average diameter: 200 nm) and morphologically close to vesicles containing viral particles or dense bodies. We also confirmed the absence of degradative autophagic vacuoles in infected cells, as we had previously described^10^. In the same area of the cytoplasm, several events of capsid envelopment in a crescent-shaped double membrane (insert b) were observed. Taken together, our results suggest a role of the autophagic machinery in the acquisition of the final envelope of HCMV.

## Discussion

In this study, the establishment of different autophagy-deficient human cell lines allowed us to confirm that autophagy exerts a proviral effect on HCMV multiplication. We had previously reported, but mainly by pharmacological approaches and by inducible invalidation of ATG16L1 gene, that inhibition of autophagy negatively impacts HCMV production^11^. It was important now to extend this study by extinction of other autophagy proteins in order to differentiate the effect of the whole autophagic process from the effect of one specific autophagy protein. Whereas ATG4B, ATG5 and ATG16L1 have a role on lipidation of LC3 and its homologs, BECN1 intervenes in two complexes regulating initiation and maturation of autophagosomes. However, it is clear that the functions of each ATG protein are not solely related to autophagy *per se*. Several ATG proteins essential for the autophagy process are also necessary for other membrane-related functions^18^. For example, UVRAG, although well known to activate autophagy, promotes entry of Vesicular stomatitis Virus (VSV) and influenza A Virus (IAV) independently of autophagy, through multiple interactions with the membrane fusion cellular machinery^30^. Similarly, it has been recently reported that ULK1 also regulates ER to Golgi trafficking and the assembly of ER exit sites^31^. The impact on HCMV multiplication was similar in all the autophagy-deficient cell lines tested and we thus concluded that most of the core autophagic machinery seems beneficial for HCMV. As we observed a decrease in LC3-II in all the deficient cell lines, we cannot exclude that the proviral role is mainly related to LC3. This effect was not limited to the HCMV laboratory adapted AD169 strain since similar results were obtained with the clinical-like TB40/E strain. Now, it will be important to pursue this study by blocking autophagosome maturation and fusion with lysosomes by using invalidation of protein expression or genes specifically required for this step. The machinery involved in this process is based on three sets of protein families: Rab GTPases, membrane-tethering complexes such as HOPS (homotypic fusion and protein sorting), and SNAREs (soluble N-ethylmaleimide sensitive factor attachment protein receptors)^32^.

In order to investigate the precise role of autophagy on HCMV multiplication, we focused on cytoplasmic viral assembly in this study, because autophagy has no significant role on HCMV viral entry, genome replication, viral protein expression and viral release. In the case of HCMV infection, the formation of vAC, which ensures the final cytoplasmic steps of the particle assembly, was described by numerous groups^2,4,6,33–35^. We confirmed the organization of vAC in concentric rings by confocal analysis and multiple co-immunostaining of cellular and viral components, as previously observed^7^ (Fig. 7B). Early endosomes were mainly located in the center of the vAC, whereas late endosomes/lysosomes (LAMP1+) were present more homogeneously inside the vAC, together with the viral tegument protein pp28. Short Golgi stacks, formed by HCMV-induced fragmentation of the Golgi apparatus, were organized in a ring-shaped structure, as previously described^35^. Moreover, we described for the first time that LC3 and two GABARAP homologs (GABARAPL1 and GATE16) were present in the vAC, mainly at the outer edge, during HCMV infection. Antibodies used against LC3 in our confocal analyses recognize the three homologs of LC3, (LC3 A, B and C) and prevented us from concluding for a specific recruitment of one of them. However, we demonstrated that LC3 puncta within the vAC were LC3-II positive vesicles by different approaches. Indeed, the main form of LC3 present in the vAC is lipidated and associated with membranes. We also confirmed that the other ATG8 homologs accumulated in the assembly compartment in their lipidated form. Colocalization of LC3 with GABARAPL1 and GATE16 (Fig. 5A,B) and partial colocalization with LAMP1 suggest that the LC3/GABARAP+ vesicles observed in the vAC could be phagophores, autophagosomes or amphisomes. Moreover, using transmission electron microscopy, we observed numerous double membrane vesicles in close proximity to enveloped viruses. The autophagic receptor p62 also accumulated during the course of infection and was recruited mainly in the periphery of the vAC and to a less extent in the vAC. We visualized vAC in different HCMV-infected autophagy-deficient cells, suggesting that autophagy is not required to initiate the formation of the vAC. Moreover, a structure we named PrAC, containing LC3 positive vesicles and fragmented Golgi stacks was observed as early as 10 hours pi and seemed to precede the formation of the vAC *per se*. Identification of the viral proteins involved in the PrAC formation are now necessary. They are probably early rather than late proteins.

HCMV uses cytoplasmic membranes to acquire its final envelope but the precise origin of these membranes is still controversial^36^ and according to Schauflinger *et al*., different types of membranes could be used to form its envelope^5^. This is in accordance with our data, which showed that in autophagy-deficient cells, viral production was clearly decreased, but less than 10 times. We think that autophagic membranes, although non-essential, could contribute to HCMV envelopment. Interestingly, our electron micrographs of HCMV-infected cells showed accumulation of autophagosomes and HCMV nucleocapsids budding into crescent-shaped double-membrane vacuoles which look like phagophores. Presence of autophagosomes in HCMV-infected cells had been previously reported by us and, in fact, as early as 1978 by Smith and de Harven^9,37^. Nucleocapsids being wrapped into cytoplasmic vesicles closely resemble images of autophagosome formation, from the elongation and curvature of the phagophore to the closure of the double membrane vacuole^5^ (Fig. 7A,C). Based on that, we purified extracellular viral particles by two different ways, and analyzed their content for ATG proteins. As anticipated in our model, we found LC3 homologs associated with purified virions but also more surprisingly p62, BECN1 and ATG5-ATG12. To exclude cellular debris, we further purified the virions on a tartrate/glycerol gradient, and we confirmed the association of ATG proteins with viral particles. Moreover, we mainly observed lipidated forms of LC3B and GABARAPL1 in virions and high levels of BECN1. We had previously observed that BECN1 is able to interact with TRS1 and IRS1^11^, which are viral proteins included in the tegument inside the viral particle. Therefore, BECN1 could be present in the tegument of the virions. The presence of p62 with purified HCMV particles could be related to its function as an autophagy receptor, since it allows recruitment of cargos into autophagosomes by interacting with ATG8 homologs. Interestingly, binding of viral components with p62 has already been reported for two viruses, Sindbis and Chikungunya, but in both cases this interaction leads to virophagy, a selective degradation of the viral proteins^38,39^. The role of p62 in the context of HCMV infection needs to be further investigated. More generally, we observed accumulation of several autophagy proteins during infection, and it would be interesting to investigate their mRNA levels. Finally, we tested the presence of the early endosomal marker EEA1 and the Golgi marker GM130 in the same conditions and observed no detectable level of EEA1 and a slight band for GM130 in purified HCMV virions. These results confirmed previous data from Cepeda *et al*., suggesting that EEA1 may not be incorporated into virions or at least that protein levels are under the detection threshold^8^. It will be interesting to investigate by super resolution microscopy and by immuno-electron microscopy where BECN1, p62, ATG5, LC3 and GABARAPs are located with respect to each other in the viral particle.

Our results are comparable with data obtained with two other human Herpesviruses, EBV and VZV, which modulate autophagy^21^. Interestingly, Nowag *et al*. showed that LC3 is associated with highly purified EBV virions^40^ and another recent study showed the co-purification of LC3 and Rab11 with extracellular VZV particles^41^. It would be interesting to seek whether other ATG proteins are associated with EBV and VZV virions, in particular LC3 homologs and BECN1. In fact, the use of autophagy proteins could be a general feature of Herpesviruses to optimize their cytoplasmic envelopment. Although both EBV and VZV use autophagy for their own profit, EBV blocks the autophagic flux^40,42^ like HCMV, whereas VZV induces a complete functional autophagy process but somehow manages to escape autophagic degradation^43^. For VZV, the authors proposed a model where autophagy plays a role in exocytosis because enveloped viral particles were found in single membrane vesicles decorated with amphisome markers^41^. However, it cannot be excluded that the virus acquires its envelope thanks to autophagic membranes since envelopment from a phagophore structure would also lead to viral particles in single membrane vacuoles (Fig. 7B). Regarding HCMV, another additional role of ATG proteins association with viral particles could be related to an improvement of its infectivity or its stability. It has been reported that the stability of influenza A extracellular viral particles is promoted by virus-induced redistribution of LC3 at the plasma membrane during infection^44^.

In conclusion, our study contributes to a better characterization of the HCMV assembly compartment. We identified novel cellular partners proposing a new function of autophagy and LC3 homologs in HCMV envelopment and thus viral replication.

## Methods

### Cells and viruses

Human foreskin fibroblasts (HFF), provided by Thomas Shenk (Princeton University, Princeton, USA), were maintained in Dulbecco minimum essential medium (DMEM) (Gibco, 41965–039) supplemented with 10% fetal calf serum (FCS). Primary human embryonic lung fibroblasts MRC5 were purchased from Biomérieux and used between passages 23 and 28 post-isolation. These cells were maintained in MEM (Gibco, 21090-055) supplemented with 10% FCS, penicillin G (100 U/ml), streptomycin sulfate (100 µg/ml), l-glutamine (1%), and nonessential amino acids (1%). Cells were cultured at 37 °C under 5% CO_2_. AD169 strain of HCMV was obtained from ATCC and was propagated in MRC5 cells as previously described^45^. TB40/E strain of HCMV was provided by Christian Sinzger (Institute for Virology, Ulm University Medical Center, Germany) and was propagated in HUVEC cells followed by amplification in MRC5 cells^46^. Starvation-induced autophagy was carried out by culturing the cells in Earle’s Balanced Salt Solution (EBSS) (Gibco, 24010-045) for 4 h before fixation and autophagic flux was monitored by addition of chloroquine (Sigma, C6628) 4 h before cell lysis. Previously described lentiviruses bearing p41-LC3 or p41-LC3 G120A were used to transduce HFF^22^.

### Autophagy-deficient HFFs

Stable HFF cell lines deficient in autophagy were established using lentiviral transduction. Lentiviruses bearing *trans*-dominant negative ATG4B (ATG4B C74A) fused to the fluorescent protein Strawberry, LC3B, and scrambled shRNA had been previously described^22^. ATG5, BECN1, ULK1 and non-target shRNA were purchased from Sigma (MISSION pLKO.1-puro shRNA lentiviruses). After transduction, ATG4B C74A HFF were sorted by flow cytometry (FACSAriaII, IPSIT, Antoine-Béclère hospital, France), while shLC3B, shSCR, shATG5, shBECN1, shULK1 and shNT-expressing HFF were selected by addition of puromycin in culture media (5 µg/ml; InvivoGen). Due to the different lentiviral vectors used, cells stably expressing the shRNA targeting ATG5 (shATG5), BECN1 (shBECN1) and ULK1 (shULK1) were compared to control cells expressing a non-target shRNA (shNT), whereas we used cells expressing a scrambled shRNA (shSCR) as a negative control to compare with cells invalidated for LC3B expression (shLC3B) cells. HFF overexpressing ATG4B-C74A (ATG4B C74A) were compared to wild type (WT) cells, because it had been described earlier that overexpression of WT ATG4B also inhibits autophagy^23^.

To study cell proliferation, cells were detached 4 days after seeding by trypsinization and enumerated by trypan blue exclusion and counting with a hemocytometer. Viability of cells infected with HCMV AD169 strain at MOI 0.01 during 4 days was assessed by MTT assay using 0.3 mg/mL thiazolyl blue tetrazolium bromide (Sigma, M5655). Absorbance was measured using the infinite f50 microplate reader (Tecan) at 550 nm with a reference wavelength at 630 nm. Each treatment was performed in triplicate.

### Antibodies

To detect HCMV-infected cells, we used mouse monoclonal antibodies directed against the viral proteins IE1 and IE2 (clone E13; Biomerieux, 11–003), tegument proteins pp65 (Novus, B051M) and pp28 (Santa Cruz, clone CH19, sc-69749). We also used a rabbit polyclonal antibody directed against the viral proteins IRS1 and TRS1 provided by Adam Geballe^47^. Additional primary antibodies used in this study included anti-SQSTM1 (p62) (Abnova clone 2C11, H00008878-M01), anti-BECN1 (BD bioscience, 612112), anti-β-actin (Merck Millipore MAB1501 clone C4), anti-LC3 (MBL-PM036 and MBL-M152-3B), anti-LC3B (Sigma L7543, used for immunoblot analysis), anti-ULK1 (Santa Cruz, H-240 sc-33182),anti-GM130 (BD transduction, 610822), anti-GABARAPL2/Gate16 (R&D, 853746), anti-GABARAPL2/Gate16 (ProteinTech, 18724-1-AP), anti-GABARAPL1 (Cell-signaling, D5R9Y), anti-LAMP1 (Cell-signaling, D2D11), anti-EEA1 (Cell-signaling, C45B10), anti-ATG5 (sigma, A0731), anti-Gag/p41 (Abcam, ab63917) and DAPI (Invitrogen, D1306). Secondary antibodies used in this study included Alexa Fluor 555 goat anti-rabbit, anti-mouse and anti-rat (Life technology, A21428 – A21424 – A21434), Alexa Fluor 488 goat anti-rabbit and anti-mouse (Jacskon ImmunoResearch, 111-545-003 and 115-545-003), Alexa Fluor 647 donkey anti-rabbit and anti-mouse (life technology, A31573 and A31571). Horseradish peroxidase (HRP)-labeled goat anti-mouse and anti-rabbit secondary antibodies were purchased from Jackson ImmunoResearch Laboratories (115–035–003, 111–035–003).

### HCMV infection and titration

HCMV suspended in serum-free DMEM, or serum-free DMEM alone (mock-infected) was adsorbed onto cells for 1 h at 37 °C at various MOI. After the inoculum was removed, the cells were maintained in DMEM containing 10% FCS and processed for the different assays at various times pi. For growth analysis, viruses were adsorbed onto cells for 2 h at 37 °C and after removal of inoculum, extracellular virus particles were inactivated using citrate buffer (40 mM citric acid, 10 mM KCl, 135 mM NaCl, pH = 3). At the indicated times, viruses were collected from cell-free supernatant fractions or infected cells and quantified as previously described^48^. The cell-associated virus was isolated through 3 rounds of freezing and thawing.

### HCMV purification

Purification of HCMV AD169 virions on glycerol-tartrate gradient was performed as described before^10^. Briefly, after ultracentrifugation of supernatants from infected MRC5 cells, virion-containing pellets were resuspended in 0.5 ml of MEM and transferred onto a preformed linear glycerol-tartrate gradient (15 to 35% Na-tartrate and 30 to 0% glycerol in 0.04% Na-phosphate). Virion fractions were harvested using syringes and needles after ultracentrifugation of the gradient. Subsequently, virions were washed and pelleted by an additional ultracentrifugation. Pellets obtained either after the first ultracentrifugation or after the whole purification process were resuspended in lysis buffer for western-blot analysis.

### Real-time PCR

To determine viral DNA levels in infected cells, cells were harvested and lysed in the presence of proteinase K and total DNA was purified using the DNeasy Blood and Tissue kit (Qiagen, 69506) according to the manufacturer’s instructions. Real-time PCR was performed using Taqman probes and primers specific for the UL123 viral gene and CXCR4 cellular gene as previously described^49^. PCR data were analyzed using CFX manager software.

### Immunoblot analysis

HFF cells were lysed in 65 mM Tris, pH 6.8, 4% SDS, 1.5% β-mercaptoethanol and held at 100 °C for 10 min. Protein extracts were resolved on SDS-PAGE gels (8% for TRS1 and IRS1, 12.5% for others) and electrotransferred onto a polyvinylidene fluoride membrane (Amersham). Membranes were incubated in blocking buffer and then probed with primary antibodies overnight. HRP-labeled antibodies were used as a secondary antibody, revealed using the ECL detection system under conditions recommended by the manufacturer (Immobilon, Millipore) and anti-actin was used to ensure equal loadings. Quantification of protein levels was performed using ImageJ software.

### Immunofluorescence analysis

Cell monolayers were washed with phosphate buffered saline (PBS) and cells were either fixed with 4% paraformaldehyde in PBS or methanol and were permeabilized using 0.2% Triton X-100 in PBS. When specified, cells were first permeabilized using buffer (0.1% Triton, 100 mM KCl, 2 mM MgCl_2_, 1 mM CaCl_2_, 1 mM Hepes) in order to deplete soluble proteins from the cytosol, and subsequently fixed with 4% paraformaldehyde in PBS. Cells were then incubated for 1 h in PBS, gelatin 0.2% supplemented with 5% of goat serum for blocking, and then with appropriate primary antibodies. The cells were washed 3 times in PBS Tween20 0.1%, and then incubated with appropriate secondary antibodies. When using rabbit polyclonal antibodies, ChromPure human IgG Fc fragment (Jackson ImmunoResearch) was added in blocking buffer, as well as with primary and secondary antibody incubations, in order to limit cross-reaction with Fc receptor-like HCMV proteins^35^. Coverslips were mounted in Glycergel (Dako, C0563) and examined using a Zeiss Axiovert 200 M epifluorescence microscope (Zeiss instruments) or a Leica TCS SP8 confocal microscope (IPSIT, cellular imaging, Châtenay-Malabry, France). Photographic images were resized, organized, and labeled using ImageJ software or LAS AF Lite.

### Electron microscopy

Cell monolayers were fixed for 30 min at 37 °C in 1% paraformaldehyde, 2.5% glutaraldehyde in 0.1 M phosphate buffer, washed, and fixed again in aqueous 1% osmium tetroxide and 1.5% potassium ferrocyanide, then progressively dehydrated in ethanol, and embedded in Epon. Ultrathin sections were cut using ultramicrotome LEICA UC6 from IMAGIF facility and processed for electron microscopy. Samples were imaged using a JEOL1400 transmission electron microscope at 80 kV and camera Orius SC1000. Micrographs were resized, organized, and labeled using ImageJ software.

### Statistics

Data are expressed as means ± standard error of the means (SEM) and were analyzed with Prism software (GraphPad) by using Student’s t test or one way analysis of variance (ANOVA) test comparisons. P values less than 0.05 were considered statistically significant. Experiments were performed a minimum of three times.

## Supplementary information

Supplementary information

Figure S2

Figure S3

Figure S4

## Data Availability

All data generated or analyzed during this study are included in this published article and its supplementary information files.
