# Supplementary material for: Human cytomegalovirus hijacks the autophagic machinery and LC3 homologs in order to optimize cytoplasmic envelopment of mature infectious particles
Source: Sci Rep. 2019 Mar 14;9:4560. doi: 10.1038/s41598-019-41029-z (PMC6418312; doi:10.1038/s41598-019-41029-z)
Supplement: Supplementary file 1 — Supplementary information [file 41598_2019_41029_MOESM1_ESM.pdf]

Supplementary Figure Legends

Human cytomegalovirus hijacks the autophagic machinery and LC3 homologs in order to optimize cytoplasmic envelopment of mature infectious particles

Clémence Taisne, Marion Lussignol, Eva Hernandez, Arnaud Moris, Lina Mouna, and Audrey Esclatine

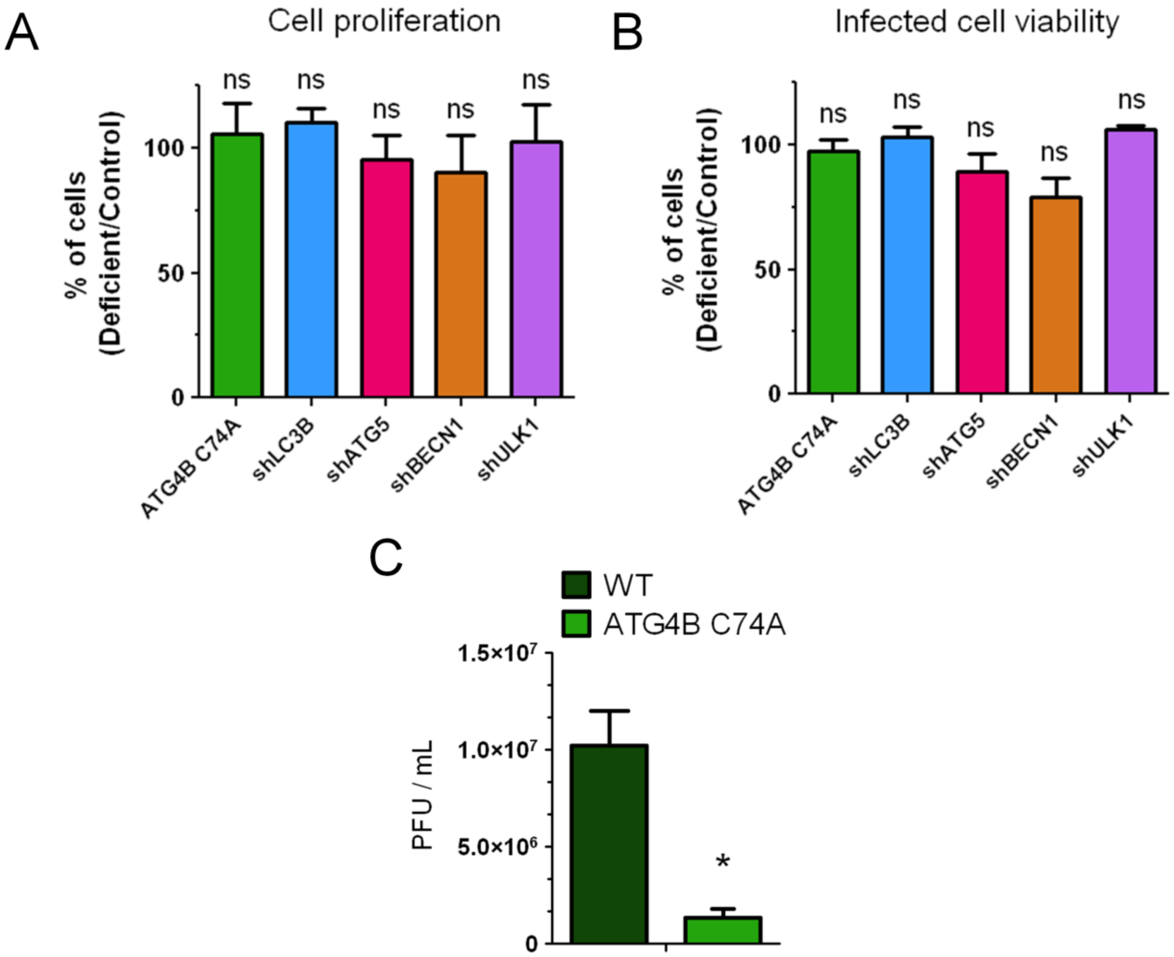

Supplementary Figure S1. Autophagy deficiency does not impact cell proliferation or infected cell viability and autophagy is beneficial for multiplication of TB40/E strain. HFF control cells (WT, shSCR, shNT) and autophagy deficient cells were compared for (A) cell proliferation, using cell enumeration by trypan blue exclusion and (B) cell viability during infection at MOI 0.01 for 4 days using MTT assay. Graphs indicate percentage of autophagy deficient cells compare to control cells. Error bars indicate SEM from three independent experiments. ns non-significant (One-way ANOVA test). (C) Human fibroblasts stably overexpressing a dominant negative of ATG4B or their wild type counterparts were infected with TB40/E strain at MOI 0.1 during 8 days and viral titers were determined in the supernatant. Error bars indicate SEM from three independent experiments. * $p < 0.05$ (Student's t test).

Supplementary figure S2. 3D Imaris reconstruction video of HCMV-infected HFF cells immunostained for LC3 (green) and for pp28 (tegument viral protein located to the AC -red). Nuclei were subsequently stained with DAPI.

Supplementary figure S3. 3D Imaris reconstruction video of HCMV-infected HFF cells immunostained for LC3 (green) and for GM130 (Golgi marker -red). Nuclei were subsequently stained with DAPI.

Supplementary figure S4. 3D Imaris reconstruction video of HCMV-infected HFF cells immunostained for LC3 (green) and for EEA1 (early endosome marker -red). Nuclei were subsequently stained with DAPI.

A

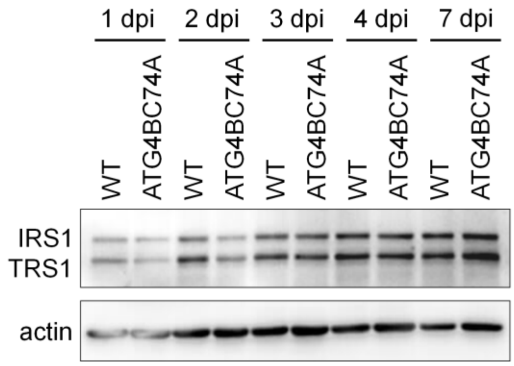

B

	1 dpi		2 dpi		5 dpi	
	WT	ATG4B C74A	WT	ATG4B C74A	WT	ATG4B C74A
IE2	0.0	0.0	0.2	0.1	0.9	0.6
IE1	0.5	0.4	0.5	0.5	1.8	1.1
IRS1	0.1	0.0	0.7	0.3	1.3	0.9
TRS1	0.1	0.1	0.8	0.3	1.2	0.8
pp28	0.0	0.0	0.2	0.1	1.5	1.0

	1 dpi		2 dpi		5 dpi	
	shSCR	shLC3B	shSCR	shLC3B	shSCR	shLC3B
IE2	0.0	0.0	0.1	0.1	0.7	0.8
IE1	0.5	0.4	0.3	0.4	1.0	0.9
IRS1	0.1	0.1	0.5	0.4	0.9	1.0
TRS1	0.2	0.1	0.6	0.5	0.6	0.9
pp28	0.0	0.0	0.0	0.1	1.2	1.3

	1 dpi		2 dpi		5 dpi	
	shNT	shATG5	shNT	shATG5	shNT	shATG5
IE2	0.0	0.0	0.1	0.1	1.0	0.8
IE1	0.4	0.4	0.5	0.7	0.9	1.1
IRS1	0.1	0.1	1.0	0.8	1.4	1.6
TRS1	0.2	0.1	1.0	0.8	1.3	1.6
pp28	0.0	0.0	0.1	0.0	1.5	1.5

	1 dpi		2 dpi		5 dpi	
	shNT	shBECN1	shNT	shBECN1	shNT	shBECN1
IE2	0.0	0.0	0.0	0.0	0.8	1.4
IE1	1.1	0.6	1.4	1.4	1.3	2.1
IRS1	0.2	0.3	1.4	1.2	1.5	1.8
TRS1	0.3	0.3	1.6	1.2	1.4	1.8
pp28	0.0	0.0	0.2	0.3	4.0	4.0

Supplementary Figure S5. Viral protein expression in autophagy deficient cell lines. *(A) Expression of IRS/TRS1 after HCMV infection of ATG4B C74A cells at MOI 0.5 for 1 to 7 days post infection (dpi). Actin was used as a loading control. (B) Quantification of 3 representative western blots of viral protein expression in autophagy deficient cell lines compared to their own control. Actin was used as a loading control.*

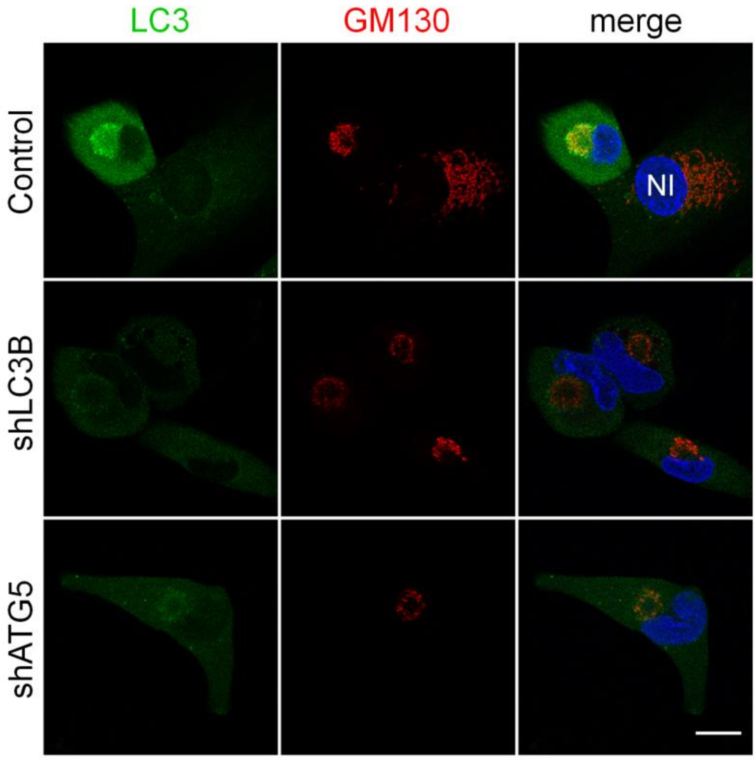

Supplementary Figure S6. vAC is present in autophagy deficient cells. Confocal images of control HFF or autophagy-deficient cells infected with HCMV AD169 strain at MOI 0.5 for 4 days and immunostained for LC3, GM130 (Golgi), DAPI (Nuclei). Note the low level of LC3 expression and the normal Golgi apparatus in the non-infected cell (NI). Scale bar = 20μm.

Figure 1 A

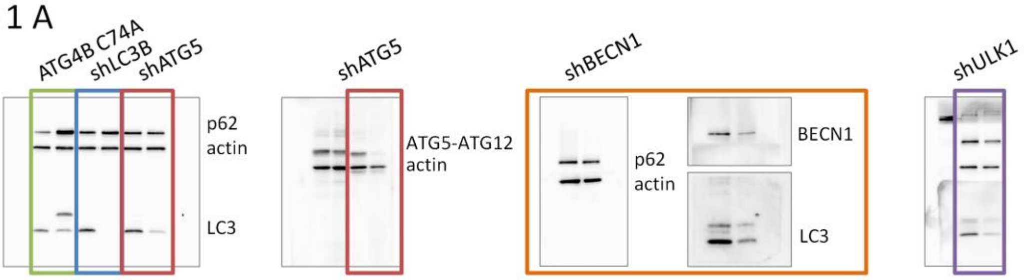

Figure 4 C

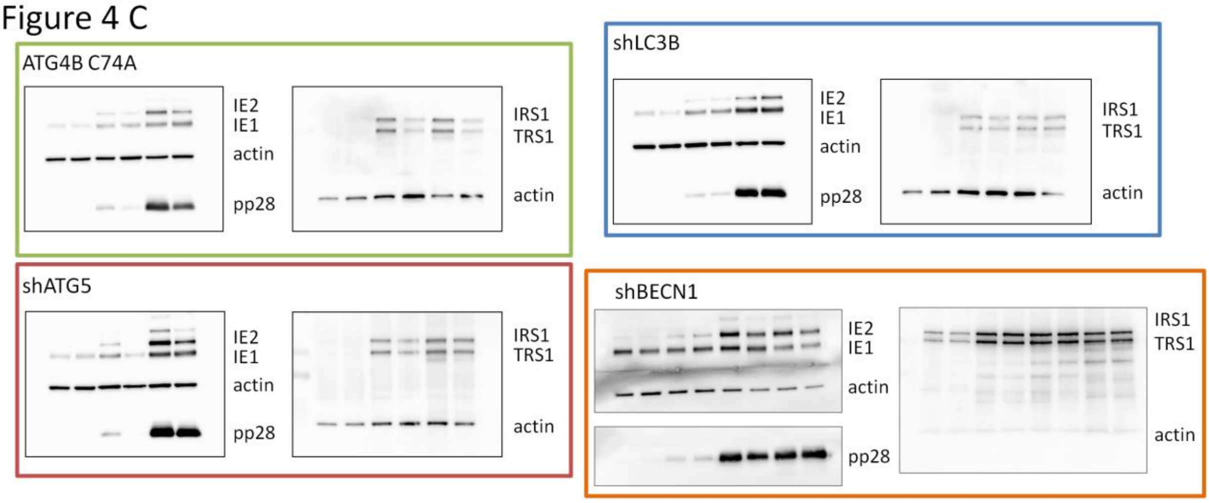

Figure 5C

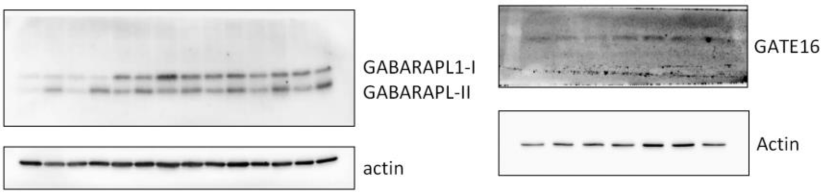

Figure 6A

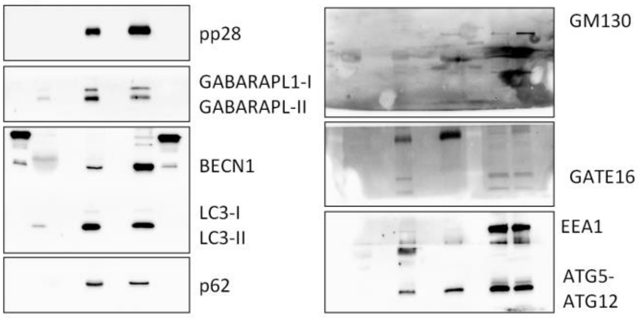

Fig.6B

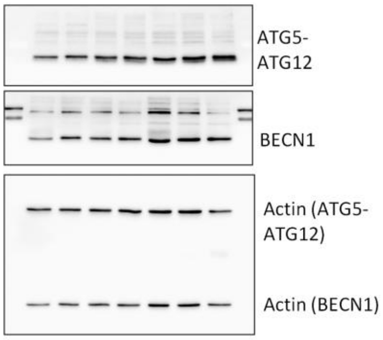

Supplementary Figure S7. Full-length western blots.
